# Offsetting pb induced oxidative stress in *Vicia faba* plants by foliar spray of chitosan through adjustment of morpho-biochemical and molecular indices

**DOI:** 10.1186/s12870-024-05227-w

**Published:** 2024-06-14

**Authors:** Reda E. Abdelhameed, Hanan Abdalla, Mohamed Abdel-Haleem

**Affiliations:** Botany and Microbiology Department, Faculty of Science, Zagazig University, Zagazig, 44519 Egypt

**Keywords:** Chitosan, Lead stress, *Vicia faba*, Antioxidant, Phenylalanine ammonia lyase, Oxidative stress, ISSR marker

## Abstract

In the course of their life, plants face a multitude of environmental anomaly that affects their growth and production. In recent decades, lead (Pb) gained an increasing attention as it is among the most significant contaminants in the environment. Therefore, in this study the effects of Pb concentrations (0, 50 and 100 ppm) on *Vicia faba* plants and attempts to alleviate this stress using chitosan (Chs; 0 and 0.1%) were performed. The results validated that with increasing Pb concentrations, a decline in growth, pigments and protein contents was observed. In the same time, a significant upsurge in the stress markers, both malondialdehyde (MDA) and H_2_O_2_, was observed under Pb stress. Nonetheless, foliar spraying with Chs improves the faba bean growth, pigment fractions, protein, carbohydrates, reduces MDA and H_2_O_2_ contents and decreases Pb concentrations under Pb stress. Pb mitigation effects by Chs are probably related with the activity of antioxidant enzymes, phenylalanine ammonia lyase (PAL) and proline. The application of Chs enhanced the activities of peroxidase, catalase and PAL by 25.77, 17.71 and 20.07%, respectively at 100 ppm Pb compared to their control. Plant genomic material exhibits significant molecular polymorphism, with an average polymorphism of 91.66% across all primers. To assess the genetic distance created among treatments, the dendrogram was constructed and the results of the similarity index ranged from 0.75 to 0.95, indicating genetic divergence. Our research offers a thorough comprehension of the role of Chs in lessening the oxidative stress, which will encourage the use of Chs in agricultural plant protection.

**Supplementary Information:**

The online version contains supplementary material available at 10.1186/s12870-024-05227-w.

## Introduction

Heavy metal pollution has increased globally with increasing economic growth, industrial productions, urban sewage, mining, and agricultural toxins [1, 2], and this pollution is emerging as a serious threat to agricultural production [3]. Because these metals enter the human body through the food chain, heavy metal pollution of the environment further jeopardises human welfare by lowering agricultural land productivity and reducing crop yields [1, 4]. Lead (Pb) is one of the most common heavy metal pollutants, highly phytotoxic and has no role in biological systems. Even though Pb is not a necessary element for plants or animals, both often absorb it readily. This metallic pollutant is released into the environment by burning fossil fuels, using agrochemicals, and manufacturing processes that produce Pb batteries, fertilizers, and insecticides [5]. It can be found in soil as a mineral as well as in the atmosphere as dusts, fumes, mists, and vapors. Pb fumes combines with rainwater to form Pb rich soil near roadsides and in turn it was taken by plants through their roots and leaves, even though it is not required as a nutrient [6].

Once inside the plant, Pb poisoning harms plants in a variety of ways from germination to yield development; its toxicity is contingent on both time and concentration. Higher exposure rates induce oxidative damage to plants as well as disturbances to the water and nutrition relationships within the plant. The primary causes of decreased rates of plant growth and seed germination during stressful conditions include Pb interference with enzyme activities, membrane damage, and stomatal closure as a result of abscisic acid induction and Pb’s negative connection with potassium in plants. As well, Pb caused structural alterations in the photosynthetic machinery and decreased green pigment biosynthesis, which delayed the metabolism of carbon [7]. Moreover, eating Pb-tainted food can cause severe health problems for people. Due to its non-biodegradable nature and easy entry into the food chain, it poses a risk to the health of both humans and animals [8]. When Pb is present, plants produce more reactive oxygen species (ROS), suffer oxidative stress, and oxidize proteins, lipids, and nucleic acids [2, 4]. Plants have a number of defense mechanisms to deal with Pb toxicity when under Pb stress. These mechanisms include decreased absorption into the cell, complex formation-mediated sequestration of Pb into vacuoles, binding of Pb by amino acids, glutathione, and phytochelatins, and production of osmolytes. Furthermore, a secondary defense mechanism involves the activation of several antioxidants to counteract the increased creation of ROS caused by Pb [9].

Maintaining sustainable agriculture is the most important issue of the century in order to feed the world’s growing population. In this respect, attempts to attenuate the stress of Pb on plants are essential especially if plant self-defense mechanisms are insufficient to mitigate Pb’s harmful effects. Recently, chitosan (Chs) has been shown in numerous studies to be able to form complexes with non-nutrient elemental ions, including heavy metals, owing to the presence of functional amino and hydroxyl groups [10, 11]. Since it is non-toxic, biodegradable, and biocompatible, this naturally occurring biopolymer has the potential to be an elicitor and biostimulant in agriculture. Via the stress transduction route, it strengthens the physiological response and lessens the negative effects of abiotic stresses [10]. Chs is obtained from chitin which is the second most prevalent naturally occurring polysaccharide and composed of repeating unit of saccharide monomer of N-acetylglucosamine. After chitin is deacetylated, Chs is obtained which is a linear polymer made of two subunits “D-glucosamine and N-acetyl-D-glucosamine” connected by glycosidic linkages [12]. According to Shamov et al. [13], the existence of this amine group makes it easier to modify structures and create functional derivatives. Applying different molecular weight Chs to a hydroponically produced edible rape plant (*Brassica rapa* L.) has been shown to mitigate the harmful effects of Cd [14]. Additionally, Chs was shown to be able to bind Ag, Zn, Cd, and Pb in rapeseed and perennial rye grass by Kamari et al. [15, 16].

Genomic information of medicinal and crop plant species has increased rapidly in the past decade by using gene sequencing and molecular markers [17]. It has been reported that different markers might reveal different classes of variation [18–20]. The advent of the polymerase chain reaction (PCR) favored the development of different molecular techniques such as random amplified of polymorphic DNA (RAPD), simple sequence repeats (SSR or microsatellite), sequence-tagged sites (STS), random amplified microsatellite polymorphism (RAMP), single nucleotide polymorphism (SNP) and inter simple sequence repeat polymorphic DNA (ISSR) [21]. The ISSR may reveal a much higher number of polymorphic fragments per primer than RAPD [22]. Assessment of genetic diversity is one of the main applications of ISSR markers [23, 24].

Faba bean (*Vicia faba* L.) is the third- ranking among feed grain legume and it is an annual herb that is a member of the Leguminosae (Fabaceae) family [25, 26]. It is one of the most significant crops grown in Egypt and the largest winter legume crop produced globally [27]. It sustains agriculture by fixing atmospheric nitrogen to improve soil fertility. Faba beans offer a consistent source of plant protein for humans and animals that can partially replace animal protein and help to reduce the negative environmental effects of consuming animal protein [28, 29]. Given the numerous sources of Pb pollution that are necessary for modern human existence and the negligible likelihood that Pb contamination will decrease anytime soon [30, 31], as well as the fact that plants growing by roadsides are exposed to high Pb levels due to their close proximity to cars that burn leaded fuel, the aim of the current investigation was to evaluate the impacts of foliar spray with various concentrations of Pb on the growth and physio-biochemical parameters of faba bean plants. Additionally, the potential advantages of utilizing Chs were examined through the assessment of antioxidant enzyme activity and genetic variations using ISSR markers, aiming to identify genetic markers associated with oxidative stress tolerance and assess the genetic response of faba bean plants to heavy metal stress induced by Pb and Chs exposure.

## Materials and methods

### Plant materials and treatment pattern

The experimentation was conducted in the Botany and Microbiology Department, Faculty of Science, Zagazig University. Seeds of faba bean were obtained after permission from the Crop Institute, Agricultural Research Center, Giza, Egypt, then surface sterilization of the seeds was done with 1% (w/v) sodium hypochlorite (NaOCl) for 5 min. Sterilized seeds were sown in plastic pots filled with 5 kg soil. After two weeks, three Pb concentrations were administered (0, 50 and 100 ppm lead acetate) and Chs with 0.1% was foliar sprayed in three times at 3-day intervals. The experiment consisted of six treatments (each with three replications), three levels of Pb (0, 50 and 100 ppm) and two levels of Chs (0 and 0.1%) (Table 1). Plants were picked up after 30 days from Pb and Chs application to assess morpho-biochemical and molecular indices.

**Table 1:** Treatments used in this study

T1^st^	T2^nd^	T3^rd^	T4^th^	T5^th^	T6^th^
Control	0.1% Chs	50 ppm Pb	0.1% Chs + 50 ppm Pb	100 ppm Pb	0.1% Chs + 100 ppm Pb

### Measurement of morphological parameters

*Vicia faba* plants, after being gathered, were cleaned with tap water to get rid of any remaining soil particles. Plant morphological characteristics were measured and documented, including shoot and root lengths as well as fresh and dry weights of the shoot and root. To test the aforementioned qualities, a random sample of three plants was selected from each treatment. Samples were weighed individually for both fresh and dry weights (FW and DW). For DW measurements, samples were stored in the oven for 72 h at 60 °C. Additionally, the number of leaves and branching was noted.

### Measurement of biochemical traits

#### Chlorophyll a, b and carotenoid content

By extracting 100 mg of fresh faba bean leaves in 85% v/v cold acetone, quantities of carotenoids and chlorophyll (Chl) were extracted. The supernatant was collected by centrifuging the mixture at 7500 g for 10 min, and the absorption was measured using UV-Vis spectrophotometry (RIGOL, Model Ultra-3660) at 663, 644, and 452.5 nm [32]. Utilizing formulas designated by Lichtenthaler and Wellburn [33], the contents of photosynthetic pigments (carotenoids, Chl a, and b) were determined as and their concentration was expressed as mg/g FW.

### Total soluble protein content

Fresh faba bean leaves (0.25 g) were ground in 5 mL of potassium phosphate buffer pH 7.0 to assess the total soluble protein content. The homogenate was subsequently centrifuged for 30 min at 4 °C and 4500 g. Combined with a freshly prepared alkaline copper solution, 1 mL of solubilized protein was left for 10 min, followed by the addition of Folin-Ciocalteau reagent for 30 min and the absorbance was measured at 700 nm using UV-Vis spectrophotometry (RIGOL, Model Ultra-3660) [34]. The protein concentration was calculated using bovine serum albumen as a standard then expressed as mg/g FW.

### Carbohydrates content

To quantify the amount of carbohydrates based on Dubois et al. [35], 100 mg of dried faba bean leaves were extracted with 2.5 N HCl. Then, 5% phenol and concentrated H_2_SO_4_ were combined with one mL of the extract, and the absorbance was measured at 490 nm using UV-Vis spectrophotometry (RIGOL, Model Ultra-3660). The concentration of carbohydrate was expressed as mg/g DW using glucose as a standard curve.

### Hydrogen peroxide (H_2_O_2_) and lipid peroxidation contents

Velikova et al. [36] provided the methodology for measuring the H_2_O_2_ concentration of faba bean leaves. In summary, 0.25 g of fresh leaves were homogenized with 5 mL of trichloroacetic acid (TCA, 0.1% w/v), and the mixture was centrifuged at 7500 g for 15 min. At that point, 0.5 mL of the supernatant was combined with 0.5 mL of potassium phosphate buffer and 1 mL of potassium iodide (1 M). Finally, the absorbance of the mixture was recorded at 390 nm and H_2_O_2_ was articulated as mg/g FW.

Heath and Packer’s [37] method of measuring malondialdehyde (MDA) level was used to determine the degree of lipid peroxidation. After homogenizing fresh faba bean leaves in 0.1% TCA, the leaves were centrifuged for 15 min at 7500 g. The supernatant was combined with 0.5% thiobarbituric acid in a 20% TCA solution. The finished mixture was heated for 30 min at 95 °C and then was cooled and centrifuged for 15 min at 7500 g. Using UV-Vis spectrophotometry (RIGOL, Model Ultra-3660), the absorbance was measured at 532 and 600 nm, and the MDA content was computed and represented as nmol/g FW.

### Proline content

Using the Bates et al. [38] method to measure proline concentration, 0.25 g of faba bean leaf samples were homogenized in 10 mL of aqueous sulfosalicylic acid (3%) in an ice bath and the mixture was centrifuged (7500 g, 4 °C) to obtain the supernatant. In summary, 2 mL acid ninhydrin and 2 mL glacial acetic acid were added to 2 mL supernatant, thoroughly mixed, and incubated at 100 °C for one hour. Once the reaction was halted in an ice bath, 4 mL of toluene were added and well combined. Following the measurement of the mixture absorbance at 520 nm, the concentration was determined as µmol/g FW.

### Antioxidant enzymes activity

Samples of faba bean fresh leaves (1 g) under different treatments were extracted using 10 mL 50 mM potassium phosphate buffer pH 7.0 containing 0.1 mM EDTA and 1% polyvinyl pyrrolidone [39]. At 4 °C, the homogenate was centrifuged for 10 min at 7500 g, after centrifugation, the supernatant was collected to evaluate the activity of catalase (CAT) and peroxidase (POX). By measuring H_2_O_2_ consumption at 240 nm, CAT (EC 1.11.1.6) activity was ascertained [40]. Additionally, pyrogallol was used as the substrate, and the increase in absorbance at 470 nm was used to measure POX (EC 1.11.1.7) activity [41].

### Phenylalanine ammonia lyase (PAL) activity

According to McCallum and Walker [42], PAL (EC 4.3. 1.24) activity of faba bean enzyme extract was measured using phenylalanine as a substrate. L-phenylalanine was added to a 0.06 M borate buffer and crude enzyme to start the reaction. For 30 min, tubes were incubated and at 290 nm, the absorbance was measured to assess the PAL activity.

### Pb concentration

After washing the plant sample under tap water and drying it for 48 h at 60°C in the oven, the concentration of Pb in the shoots was determined. The dry sample was weighed and then crushed with a mortar and pestle to a fine powder, and mixed using a wet digestion process with a concentration of H_2_SO_4_: H_2_O_2_ (2:1 v/v) [43]. The Pb concentration was determined spectrophotometrically at the Faculty of Veterinary Medicine’ Central Lab, Zagazig University, using Inductively Coupled Plasma Spectrometry (ICPS) and was calculated [44] according to the following equation:

$$\text{C}\text{o}\text{n}\text{c}\text{e}\text{n}\text{t}\text{r}\text{a}\text{t}\text{i}\text{o}\text{n}=\frac{\text{I}\text{C}\text{P}\text{S} \,\text{r}\text{e}\text{a}\text{d}\text{i}\text{n}\text{g} \times \text{t}\text{o}\text{t}\text{a}\text{l} 	 \text{ }\text{v}\text{o}\text{l}\text{u}\text{m}\text{e} \text{ }\left(\text{m}\text{L}\right)}{\text{W}\text{e}\text{i}\text{g}\text{h}\text{t} \text{ }\text{o}\text{f}\, 	 \text{s}\text{a}\text{m}\text{p}\text{l}\text{e}\, \left(\text{g}\right)}$$

### Measurement of molecular indices

#### DNA based molecular marker

##### DNA extraction

Genomic DNA was extracted and purified from the young leaves of the faba bean plants using the genomic plant DNA extraction Kit, following the manufacturer’s protocol (Intron biotechnology, Korea).

### ISSR fingerprinting

Ten ISSR primers were screened for DNA fingerprinting; only 6 primers were amplified across all species of which were polymorphic. The name, sequence, and annealing temperature of the primers are given in Table 2. In the amplification reactions of genomic DNA, a total of 20 µL reaction mix was prepared (10 µL Thermo Scientific Maxima Hot Start PCR Master Mix (2X), 2 µL primer, 1 µL template DNA and 7 distilled water). Amplification conditions were “Initial strand separation step of 5 min at 94°C followed by 35 cycles each consisting of a denaturing step of 1 min. at 94°C, annealing step of 1 min. at 57°C and an extension step of 1.30 min. at 72°C. The last cycle was followed by 5 min. extension at 72°C to allow complete extension of the PCR products with a final hold at 4°C till electrophoresis. 20 µL of the PCR-products of each primer loaded into the wells of agarose gel (1%). The ISSR fingerprinting was visualized and photographed using a Gel Documentation and Analysis Systems and illustrated obviously using matrix plot. All reactions were repeated at least twice to check the reproducibility of the banding patterns [45].

**Table 2:** The code and sequences of the ten ISSR primers, number of polymorphic (unique, non-unique bands) and monomorphic bands generated by the ISSR analysis the studied samples

No	Primes and number of their amplification DNA bands					Types of amplified bands					% of Polymorphism
	Primer code	Primer sequence	Amplicon lengths (bp)	Total no. of DNA bands	% of amplified bands	Monomorphic bands	Polymorphic bands			Total no. of mono- and polymorphic bands	
							Unique band	Non-unique	Polymorphic bands		
1	ISSR-1	(AC)8GG	300–800	18	14.75	1	1	11	12	13	92.3
2	ISSR-2	(CTC)6	200–800	21	17.21	1	2	4	6	7	85.7
3	ISSR-3	(TG)8AA	100–900	30	24.59	1	0	7	7	8	87.5
4	ISSR-4	(AG)8T	300–900	17	13.93	0	3	10	13	13	100
5	ISSR-5	(TG)8AA	300–700	18	14.75	0	3	11	14	14	100
6	ISSR-6	(CA)8T	200–700	18	14.75	0	3	6	9	9	100
Total DNA bands				122	100	3	12	49	61	64	95.31

### Data analysis of ISSR

For data analysis, each band generated by ISSR was considered as single gene locus and amplified DNA products were scored based on the presence (1) or absence (0) of a DNA band for each primer. Polymorphic DNA bands (unique and non-unique) and monomorphic bands were also scored, and DNA polymorphism generated by ISSR were estimated based on the number of polymorphic (unique and non-unique DNA bands), and monomorphic DNA bands, and the molecular sizes of bands as well as band intensities for each sample.

### Clustering dendrogram analysis, scattering diagram, and genetic similarity matrix

The genetic relationships were assessed based ISSR fingerprinting. The clustering of the examined accessions was performed based on squared Euclidean distance to produce a distance tree using the PAST-pc Version 4.22 developed by Hammer et al. [46]. In addition, a Principal Component Analysis (PCA) in the PAST-pc was used to construct scattering diagram of the examined samples [46, 47]. Genetic similarity among species was calculated according to Dice similarity coefficient [48, 49].

### Statistical data analysis and figures

A two-way factorial (3 × 2) with three replications was used for data analysis. Using SPSS software, the variables were compared across treatments, and at the 95% probability level, the means of each treatment were examined using one-way ANOVA and Duncan’s multiple range tests. Mean ± standard error (SE) was the data’s expression.

## Results and discussion

### Morphological parameters of chs-treated faba bean plants under pb stress

**Fig. 1:**
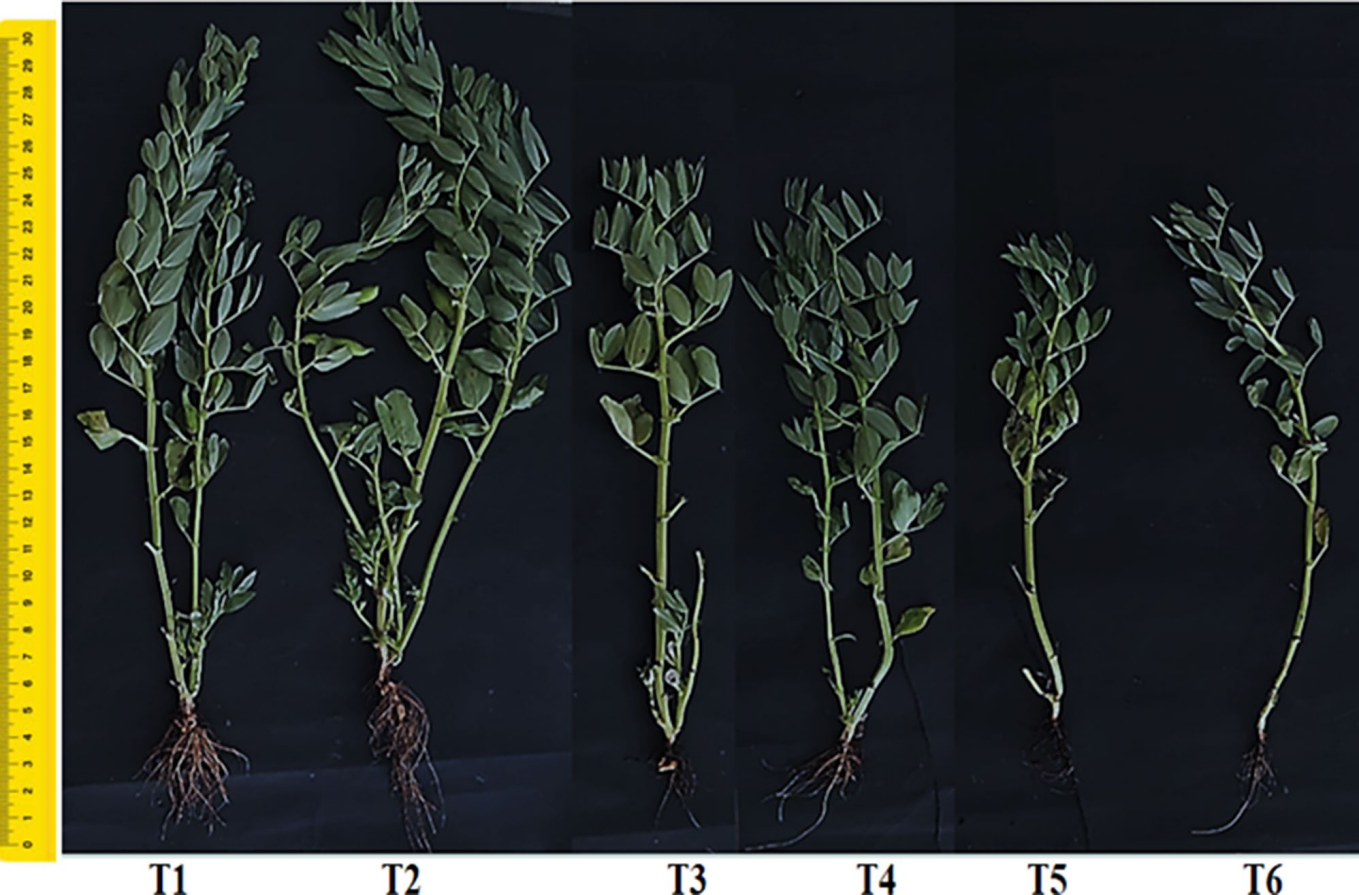
Effect of Chs application on *V. faba* plants grown under different Pb concentrations. **T1**: Control, **T2**: Chs, **T3**: 50 ppm Pb, **T4**: Chs + 50 ppm Pb, **T5**: 100 ppm Pb, **T6**: Chs + 100 ppm Pb

The effect of Chs (0.1%) and Pb concentrations (50 and 100 ppm) on growth attributes of faba bean plants was shown in Table 3; Fig. 1. The results showed that the growth parameters were significantly (*p* < 0.05) inhibited with Pb exposure; where, the shoot length decreased by 18.86 and 24.53%, and shoot FW declined by 33.64 and 62.35% under 50 and 100 ppm Pb, respectively, over their respective control ones. Additionally, the decline was greater at the highest (100 ppm) than at the lowest (50 ppm) Pb concentration. Similar to this, rising Pb concentrations in tomatoes had a detrimental effect on the fresh and dry biomass of the roots, shoots, and leaves [50]. Furthermore, Lamhamdi et al. [51] reported that there is a definite growth inhibition in spinach plants subjected to 15 mM Pb, where spinach FW and DW decreased by 28% and 29%, respectively, when compared with controls and they explained that these symptoms can be essentially attributed to macro-elements shortage (K, P, Ca and Mg), where their uptake was inhibited under Pb exposure. Furthermore, it’s possible that Pb’s inhibitory effects on growth and biomass production are due to effects on plant metabolic pathways [52]. Based on studies conducted by Mukherji and Maitra [53] and Burzynski and Jakob [54], Pb-induced promotion of indole-3-acetic acid (IAA) oxidation is the main source of cell growth inhibition. Additionally, as shown by an Avena coleoptile experiment [55], Pb interferes with auxin-regulated cell elongation.

**Table 3:** Effect of Chs application on growth parameters of *V. faba* plants under different Pb concentrations

Parameters treatments	Shoot length (cm)	Root length (cm)	Shoot FW (g)	Root FW (g)	Shoot DW (g)	Root DW (g)	Leaves number	Branching number
T1	53 ± 1.402**b**	15 ± 0.391**b**	53.5 ± 1.415**b**	5.05 ± 0.134**b**	7.603 ± 0.228**b**	1.526 ± 0.0403**c**	36 ± 0.952**b**	3 ± 0.079**b**
T2	70 ± 1.851**a**	20 ± 0.529**a**	61.9 ± 1.637**a**	9.11 ± 0.241**a**	11.48 ± 0.344**a**	2.039 ± 0.0539**a**	45 ± 1.191**a**	4 ± 0.105**a**
T3	43 ± 1.131**d**	12 ± 0.317**c**	35.5 ± 0.939**d**	3.85 ± 0.102**d**	5.895 ± 0.176**c**	1.2259 ± 0.032**d**	24 ± 0.635**d**	2 ± 0.059**c**
T4	48 ± 1.269**c**	14 ± 0.371**b**	43.31.1456**c**	4.43 ± 0.117**c**	6.261 ± 0.187**c**	1.9048 ± 0.051**b**	31 ± 0.821**c**	3 ± 0.079**b**
T5	40 ± 1.058**d**	10 ± 0.264**d**	20.14 ± 0.53**e**	2.80 ± 0.074**e**	3.78 ± 0.1134**e**	0.9762 ± 0.0258**e**	19 ± 0.502**e**	2 ± 0.053**c**
T6	44 ± 1.164**cd**	12.5 ± 0.331**c**	33.00 ± 0.87**d**	3.10 ± 0.082**e**	4.764 ± 0.143**d**	1.2614 ± 0.033**d**	25 ± 0.661**d**	2 ± 0.053**c**

Results are the mean of three replicates ± SE. Bold different letters in the same column indicate significant differences between the treatments and control plants (*p* < 0.05) as measured by Duncan test. T1, T3 and T5 represent *V. faba* plants grown under 0, 50 and 100 ppm Pb concentrations, while T2, T4 and T6 represent *V. faba* plants foliar sprayed by Chs under 0, 50 and 100 ppm Pb concentrations. FW: fresh weight, DW: dry weight

Noticeably, the growth of faba bean plants was significantly impacted by the application of Chs (Table 3). Observation of growth parameters in this table indicated that application of Chs was able to increase the percentage of shoot length, FW and DW by 32.07, 15.7 and 50.9% respectively compared to control. Under Pb stress, faba bean plants treated with Chs improved the shoot length by 11.62 and 10% at 50 and 100 ppm Pb in comparison to Pb-stressed plants respectively. Our findings concur with those of Suptijah et al. [56] and Chen et al. [57], who reported that Chs spraying had the greatest impact on all plant growth parameters, including height, the number of branches and leaves, the length and width of the leaves, and the FW and DW of the plants. Also, foliar application of Chs (0.2–0.4 g/L), increased plant growth attributes in two species of sweet basil (*Ocimum ciliatum* and *O. basilicum*) as stated by Pirbalouti et al. [58]. Sheikha and AL-Malki [59] found that the length and weight of the bean (*Phaseolus vulgaris*) shoot and root were increased with Chs application. According to Guan et al. [60], Chs increases the availability and uptake of water and other necessary nutrients by modifying the osmotic pressure of individual cells, hence stimulating plant development. Enhancing antioxidant levels and enzyme activity also helps decrease the build-up of damaging free radicals (ROS). According to some studies [61, 62], applying Chs improved the transportation of nitrogen in the leaves, which helped plants grow and develop. It also increased the activity of key enzymes involved in nitrogen metabolism, such as glutamine synthetase, and nitrate reductase. Moreover, according to Uthairatanakij et al. [63], it was found that Chs may stimulate the synthesis of gibberellins, a plant hormone, and encourage growth and development *via* a signalling pathway linked to auxin biosynthesis.

#### Effect of chs application on photosynthetic pigments of faba bean plants under pb stress

It is impossible to exaggerate the significance of Chl content in determining a plant’s ability to participate in photosynthetic activities [64]. Furthermore, carotenoids are an important type of endogenous antioxidant pigment that help to quench ROS and so stop lipid membranes from peroxidizing. Both Chl and carotenoid pigments can be used to notice different stages of plant performances [65]. The negative effects that Pb has on faba bean vegetative growth mainly result from its effect on photosynthetic pigments. According to the findings listed in Table 4, both Pb concentrations exhibited inhibiting effects on the faba bean plants’ Chl and carotenoids. Table 4 shows that at 50 and 100 ppm Pb treatment, the content of Chl a and b fell considerably (*p* ≤ 0.05) compared to the control by 39.71, 68.57%, and 40.2, 63.5%, respectively. The total Chl (Chl a + b) and carotenoid content also declined significantly by 39.8, 66.96%, and 39.9, 56.9%, respectively. Our findings concurred with those reported for *Jatropa curcas* and *Coronopus didymus* [66, 67]. This could be because Pb inhibits the processes that negatively affect plant vegetative growth; disruption of the ultrastructure of the chloroplast, obstruction of electron transport, inhibition of the Calvin cycle enzymes, reduced uptake of vital elements like Mg and Fe, and induced CO_2_ deficiency as a result of stomatal closure [9]. Additionally, it prevents the action of essential enzymes involved in the manufacture of Chl, such as protochlorophyllide reductase and aminolevulinate dehydratase, while it also stimulates the activity of chlorophyllase [68, 69].

**Table 4:** Effect of Chs application on pigment fractions (µg/mg FW) of *V. faba* plants under different Pb concentrations

Parameters treatments	Chl a	Chl b	Total Chl	Carotenoids	Total pigments
T1	1.677 ± 0.044**b**	0.762 ± 0.021**b**	2.440 ± 0.064**b**	1.288 ± 0.034**b**	3.728 ± 0.0986**b**
T2	2.318 ± 0.061**a**	1.255 ± 0.033**a**	3.573 ± 0.094**a**	1.928 ± 0.051**a**	5.501 ± 0.1455**ab**
T3	1.012 ± 0.0267**c**	0.455 ± 0.012**c**	1.468 ± 0.038**c**	0.774 ± 0.021**c**	2.242 ± 0.0593**c**
T4	1.614 ± 0.042**b**	0.734 ± 0.019**b**	2.348 ± 0.062**b**	1.216 ± 0.032**b**	3.565 ± 0.0943**b**
T5	0.528 ± 0.0139**e**	0.278 ± 0.007**e**	0.806 ± 0.021**e**	0.554 ± 0.014**d**	1.361 ± 0.0361**e**
T6	0.753 ± 0.0199**d**	0.389 ± 0.011**d**	1.143 ± 0.031**d**	0.701 ± 0.0185**c**	1.843 ± 0.0487**d**

Results are the mean of three replicates ± SE. Bold different letters in the same column indicate significant differences between the treatments and control plants (*p* < 0.05) as measured by Duncan test. T1, T3 and T5 represent *V. faba* plants grown under 0, 50 and 100 ppm Pb concentrations, while T2, T4 and T6 represent *V. faba* plants foliar sprayed by Chs under 0, 50 and 100 ppm Pb concentrations

Clearly, it is evident that applying Chs greatly reduced the impact of Pb on pigment fractions, demonstrating the tremendous improvement Chs had on these measured variables. Chs application to faba bean plants increased Chl a (38.1%), Chl b (64.6%) and carotenoids (49.6%) in comparison to their respective controls in non-Pb stressed plants **(**Table 4**)**. Also, under Pb stress (100 ppm), Chs increased Chl a (42.6%), Chl b (39.9%) and carotenoids (26.5%) compared to 100 ppm Pb stressed plants only. An increase in the Chl content in plants has been confirmed by Dzung et al. [70] and Salachna and Zawadzińska [71] who reported that spraying of coffee and corm seedlings with Chs solutions enhanced the content of Chl and carotenoids in leaves in comparison to the control. Related study by Khan et al. [72] reported that the application of Chs increased photosynthesis in the leaves of soybean. Moreover, foliar application of Chs enhanced the Chl content under Ni stress [69] and Cd [73]. The increase of the Chl content as a result of application of Chs may be caused by plants’ enhanced uptake of nutrients, which occurred in the study by Nguyen Van et al. [74] on coffee seedlings where the authors demonstrated that after spraying the seedlings with Chs, an increase of the content of nitrate, phosphorus and potassium in leaves was observed. Moreover, Mukhtar Ahmed et al. [75] reported that two mechanisms were responsible for the positive effects of Chs on the content of Chl: suppression of the transcript level of chlorophyllase, a component of the catabolic pathway of Chl, and stimulation of the expression of genes involved in the biosynthesis pathway of Chl.

#### Changes in protein and carbohydrate contents in faba bean plants as affected by Chs application under normal and Pb stress condition

**Fig. 2:**
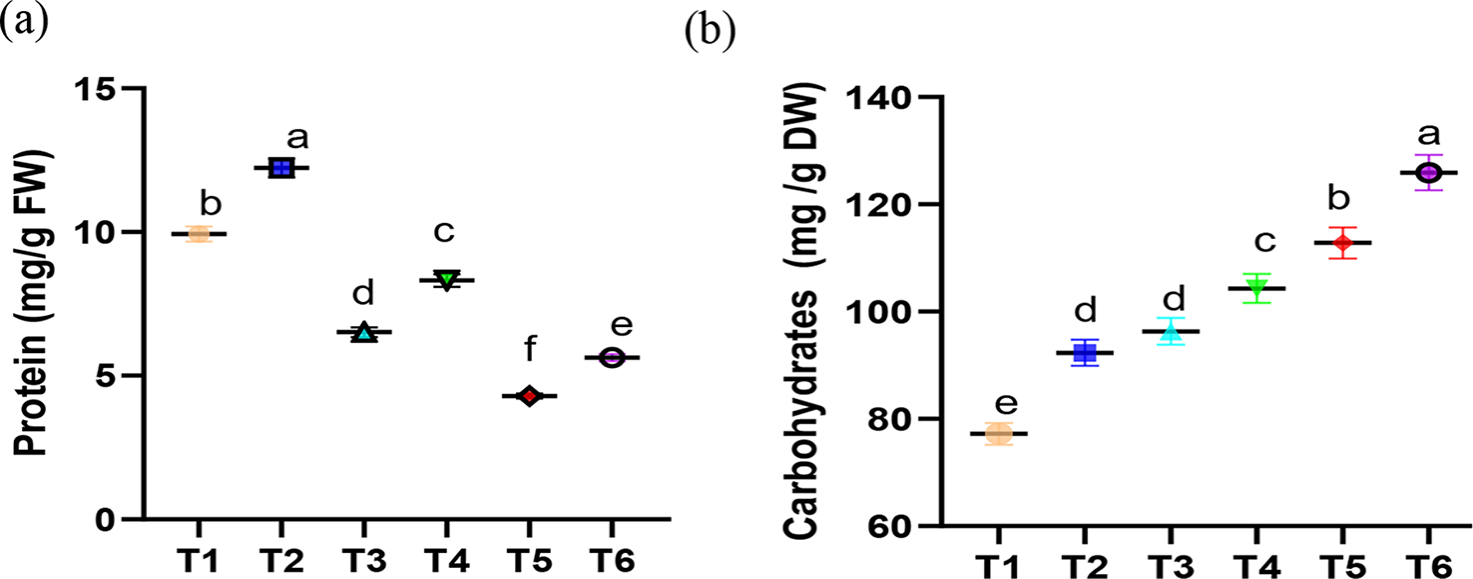
Effect of Chs application on (**a**) protein and (**b**) carbohydrates contents of *V. faba* plants grown under different Pb concentrations. T1, T3 and T5 represent *V. faba* plants grown under 0, 50 and 100 ppm Pb concentrations, while T2, T4 and T6 represent *V. faba* plants foliar sprayed by Chs under 0, 50 and 100 ppm Pb concentrations. Results are the mean of three replicates ± SE. Different letters above bars indicate significant differences between the treatments and control plants (*p* < 0.05) as measured by Duncan test

In this experiment, faba bean plants under Pb stress had lower protein content (Fig. 2a). Similar results were obtained by Sidhu et al. [67] who discovered a slight decrease in protein content in *Coronopus didymus* L after exposure to 2900 mg kg^− 1^ Pb. Related study by Bharwana et al. [76] demonstrated that the addition of Pb at both 50 and 100 µM to nutrient solution significantly reduced soluble protein content in both roots and leaves of the cotton plants. This observation could be explained by Pb-induced protein degradation, a reduction in protein synthesis during Pb stress, or by the breakdown of proteins by protease activity, which raises the degree of protein denaturation. Additionally, it has been shown that Pb stress in plants causes the production of ROS, which directly alter and modify proteins by oxidizing side chains of amino acids [77], potentially leading to protein fragmentation. Furthermore, *J. curcas* [66] and *Ceratophyllum demersum* [77] have previously been found to exhibit decreased protein content in response to Pb. However, the application of Chs significantly increased the protein content in faba bean plants under normal condition and Pb stress. Similarly, Fouda et al. [78] confirmed an increase in protein content in faba bean plants under drought stress. Strong interactions between the negatively charged phosphate groups of nucleic acids and Chs can induce specific changes in the expression and function of proteins implicated in the stress response [79]. The current findings are consistent with earlier research on the impact of Chs on *Curcuma longa* [80].

Plants must regulate their sugar metabolism in order to adapt to environmental stressors [81]. According to our findings, tissues of faba bean plants accumulated carbohydrates at both 50 and 100 mM Pb concentrations (Fig. 2b). These results were consistent with earlier research, which suggested that under Pb stress, *Coronopus didymus* [67] and *Brassica campestris* [82] had higher carbohydrate contents. Increased levels of carbohydrates in plant tissues have been documented in the literature when heavy metals are present. Additionally, Mahajan et al. [83] found that exposure to Cr increased the amount of carbohydrates in *Zea mays* leaves. Curiously, in both Pb-stressed and normal faba bean plants, exogenous Chs treatment enhanced carbohydrate accumulation. The buildup of carbohydrates in the leaves of creeping bentgrass was observed in a related study by Geng et al. [81], which was explained by the possibility that Chs might be converted to other sugars and pyruvate, which is involved in the tricarboxylic acid cycle. According to Li et al. [84], many genes involved in the transport and metabolism of carbohydrates were found to be upregulated in the leaves of white clover plants treated with Chs. Sugars build up in plant cells in response to abiotic stress as crucial signaling molecules for osmolytes, energy supply, and stress signal transduction [85].

#### Oxidative damage, proline and scavenging defense enzymes of faba bean plants as affected by Chs application and Pb stress

**Fig. 3:**
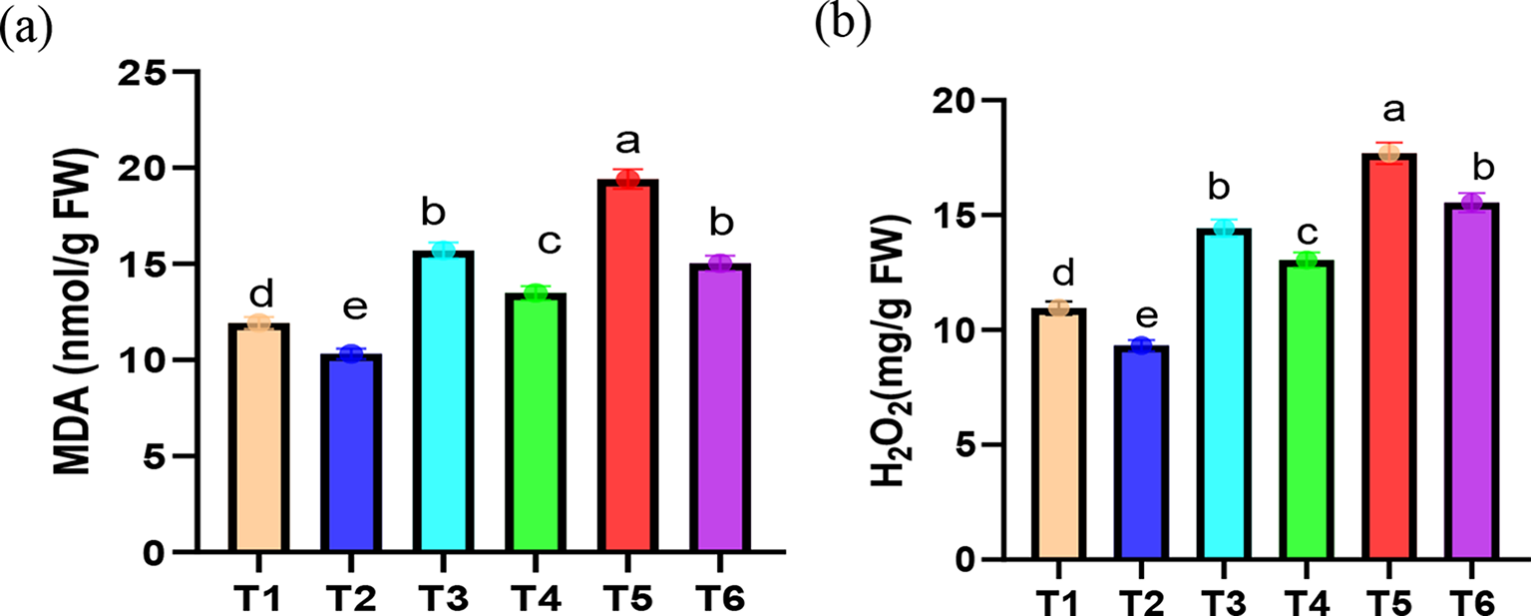
Effect of Chs application on (**a**) lipid peroxidation (malondialdehyde, MDA) and (**b**) hydrogen peroxide (H_2_O_2_) contents of *V. faba* plants grown under different Pb concentrations. T1, T3 and T5 represent *V. faba* plants grown under 0, 50 and 100 ppm Pb concentrations, while T2, T4 and T6 represent *V. faba* plants foliar sprayed by Chs under 0, 50 and 100 ppm Pb concentrations. Results are the mean of three replicates ± SE. Different letters above bars indicate significant differences between the treatments and control plants (*p* < 0.05) as measured by Duncan test

The degree of cell membrane damage can be determined using MDA, a significant byproduct of lipid peroxidation in the cell membrane [86, 87]. The effect of Chs and different Pb concentrations on generation of H_2_O_2_ and lipid peroxidation in faba bean is presented in Fig. 3. In the present study, Pb stress significantly increased H_2_O_2_ which resulted into lipid peroxidation in faba bean plants (Fig. 3a **and b**). The increase in H_2_O_2_ and MDA contents by Pb (100 ppm) was, respectively, 61.57 and 62.68% compared to the control. According to earlier study by Singh et al. [82], Pb is known to cause oxidative stress even in minute levels by causing an excess of ROS to be produced. Because of an imbalance between the production and neutralization of ROS by antioxidant systems, plant cells produce an excess of ROS as a response to heavy metal toxicity. DNA damage, mitochondrial dysfunction, and damage to cell membranes are caused by these free radicals when they react (oxidize) with different components of cells, such as proteins, lipids and fatty acids [9, 77].

Most intriguingly, foliar Chs application decreased MDA buildup in Pb-stressed plants, indicating that Chs can potentially mitigate the negative consequences of Pb stress by reducing lipid peroxidation. According to research studies by Zong et al. [73] in *Brassica rapa*, Zou et al. [88] in *T. aestivum* and Yang et al. [89] in *Malus domestica*, Chs significantly decreased the level of MDA during salt, drought, and Cd stress, respectively. The observation demonstrated that Chs may diminish the negative responses of ROS for membranes and lower the accumulation of H_2_O_2_ and MDA; maybe via stimulating the ROS forage enzymes. Chs-treated plants were able to reduce oxidative stress by producing enzyme activities in safflower (*Carthamus tinctorius* L.) and sunflower (*Helianthus annuus* L.) [90].

**Fig. 4:**
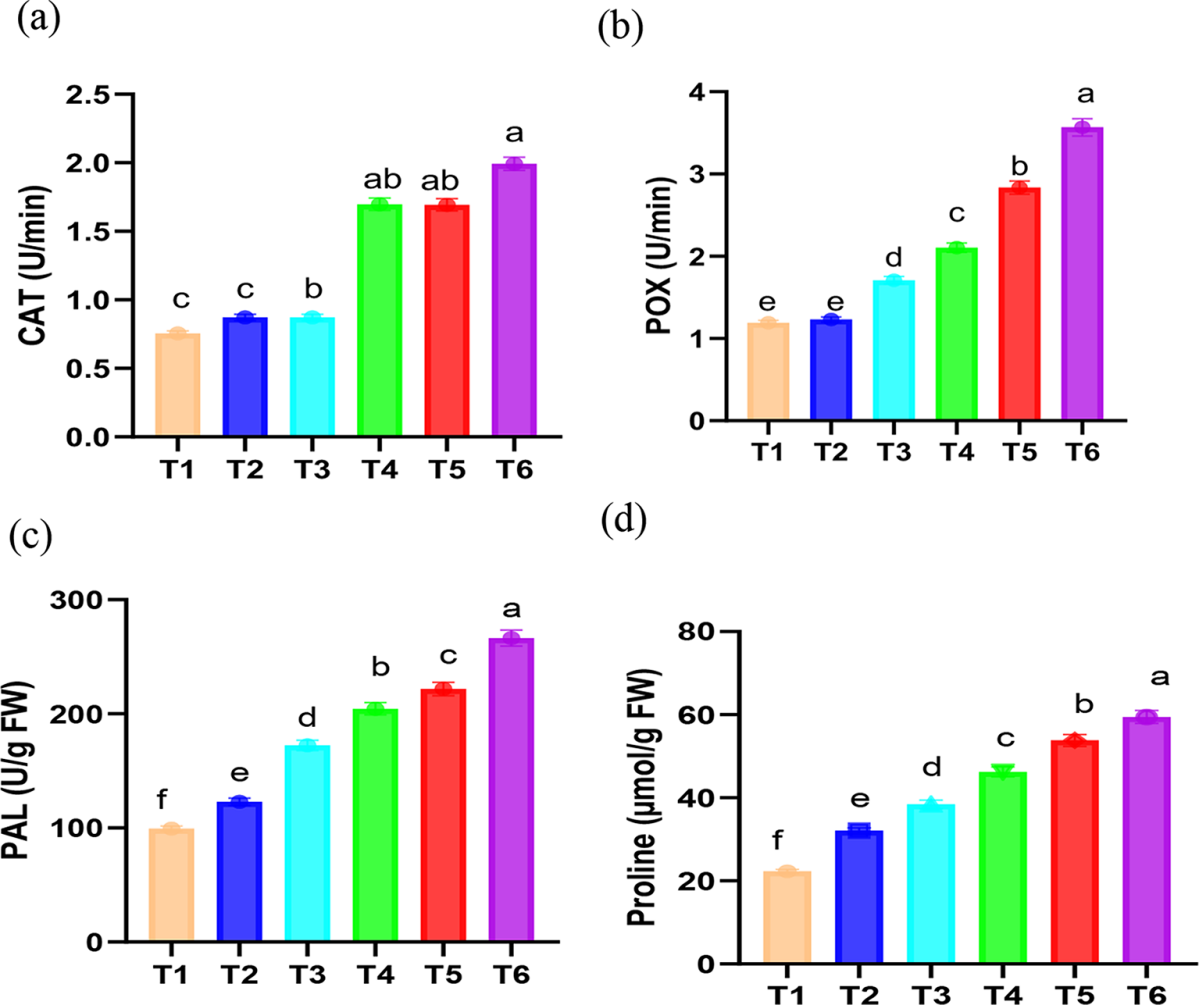
Effect of Chs application on the activity of (**a**) catalase (CAT), (**b**) peroxidase (POX), (**c**) phenylalanine ammonia lyase (PAL) and (**d**) proline contents of *V. faba* plants grown under different Pb concentrations. T1, T3 and T5 represent *V. faba* plants grown under 0, 50 and 100 ppm Pb concentrations, while T2, T4 and T6 represent *V. faba* plants foliar sprayed by Chs under 0, 50 and 100 ppm Pb concentrations. Results are the mean of three replicates ± SE. Different letters above bars indicate significant differences between the treatments and control plants (*p* < 0.05) as measured by Duncan test

Based on the current findings (Fig. 4a–c), it was shown that the exogenous application of Chs greatly enhanced antioxidative enzyme activity. CAT, POX and PAL are important enzymes which have a significant impact on the plant’s ability to adapt and, ultimately, survive under Pb stress. The present investigation revealed a noteworthy increase (*p* < 0.05) in the activity of these enzymes (Figs. 4 and 6) in response to Pb with further augumentation in their activities with Chs application in faba bean tissues. Chs application played a key role in enhancing antioxidant activity and in turn lessening MDA during Ni, Cd and drought stress as stated by Sadeghipour [69], Zong et al. [73] and Yang et al. [89], respectively. According to Xie et al. [91], Chs’s abundant active hydroxyl and amino groups, which can react with ROS to form stable and relatively nontoxic macromolecular radicals, are primarily responsible for its antioxidant properties. Ru et al. [92] suggest that Chs’s upregulated gene expression may also play a role in these properties. CAT converts H_2_O_2_ to H_2_O and O_2_ while POX breaks down H_2_O_2_ to produce phenoxy compounds, which then polymerize to produce lignans, a part of the cell wall [93]. POX is also involved in the biosynthesis of lignin, which has the potential to serve as a physical barrier against the toxicity of heavy metals [94]. According to Romanazzi et al. [95], Chs elevated PAL, which triggered the pathway leading to the synthesis of phenol. Related study by Khan et al. [72] showed transcriptional activation of gene encoding PAL was induced by Chs. The negative effects of MDA on the cell membrane can be reduced by the combined action of CAT, POX, and PAL.

To counteract the detrimental consequences of Pb stress, faba bean plants besides activating their enzymatic antioxidant defense, several osmolytic cytosolutes with coordinated antioxidant capacity such as proline was accumulated. In this investigation, it was found that Pb-stressed faba bean plants had higher proline content and the foliar application of Chs caused further rise in its content under normal and Pb stress conditions (Fig. 4d). Proline accretion has previously been shown to be a hidden predictor of stress tolerance [96]. As a stress-responsive amino acid, an accretion of proline is supposed to keep plant tissues safe from osmotic stress and this is achieved by accruing fluids that are in balance with osmoregulation, chelating and detoxifying metals, protecting enzymes, controlling cytosolic acidity, setting up the machinery for protein synthesis, and capturing ROS [97]. Our results are in line with Bistgani et al. [98] where the main causes of Chs’ compensatory action in lessening the detrimental effects of stress circumstances were proline accumulation and lipid peroxidation level reduction, which enhanced the integrity of thyme leaves’ cell membranes and stimulated osmotic adjustment. Additionally, our findings align with those of Sadeghipour [69], who noted that exogenously applied Chs had a positive effect on raising proline levels in soybeans under Ni toxicity and attributed the increased proline content under Ni toxicity to the hydrolysis of proteins caused by oxidative stress, as well as to the inhibition of proline degradation or the enhancement of de novo proline synthesis [99].

#### Pb concentration in faba bean shoots as affected by Chs application under pb stress

**Fig. 5:**
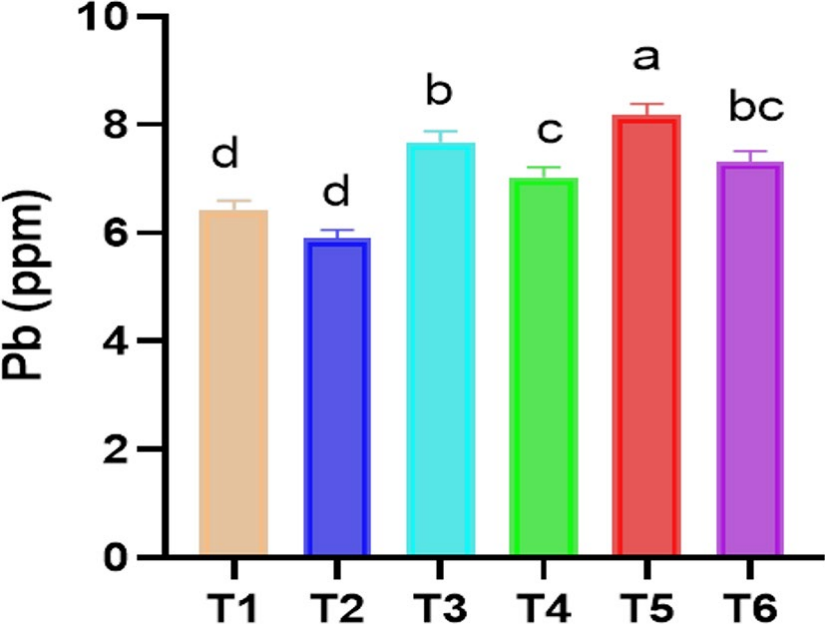
Effect of Chs application on Pb concentration in shoots *V. faba* grown under different Pb concentrations. T1, T3 and T5 represent *V. faba* plants grown under 0, 50 and 100 ppm Pb concentrations, while T2, T4 and T6 represent *V. faba* plants foliar sprayed by Chs under 0, 50 and 100 ppm Pb concentrations. Results are the mean of three replicates ± SE. Different letters above bars indicate significant differences between the treatments and control plants (*p* < 0.05) as measured by Duncan test

**Fig. 6:**
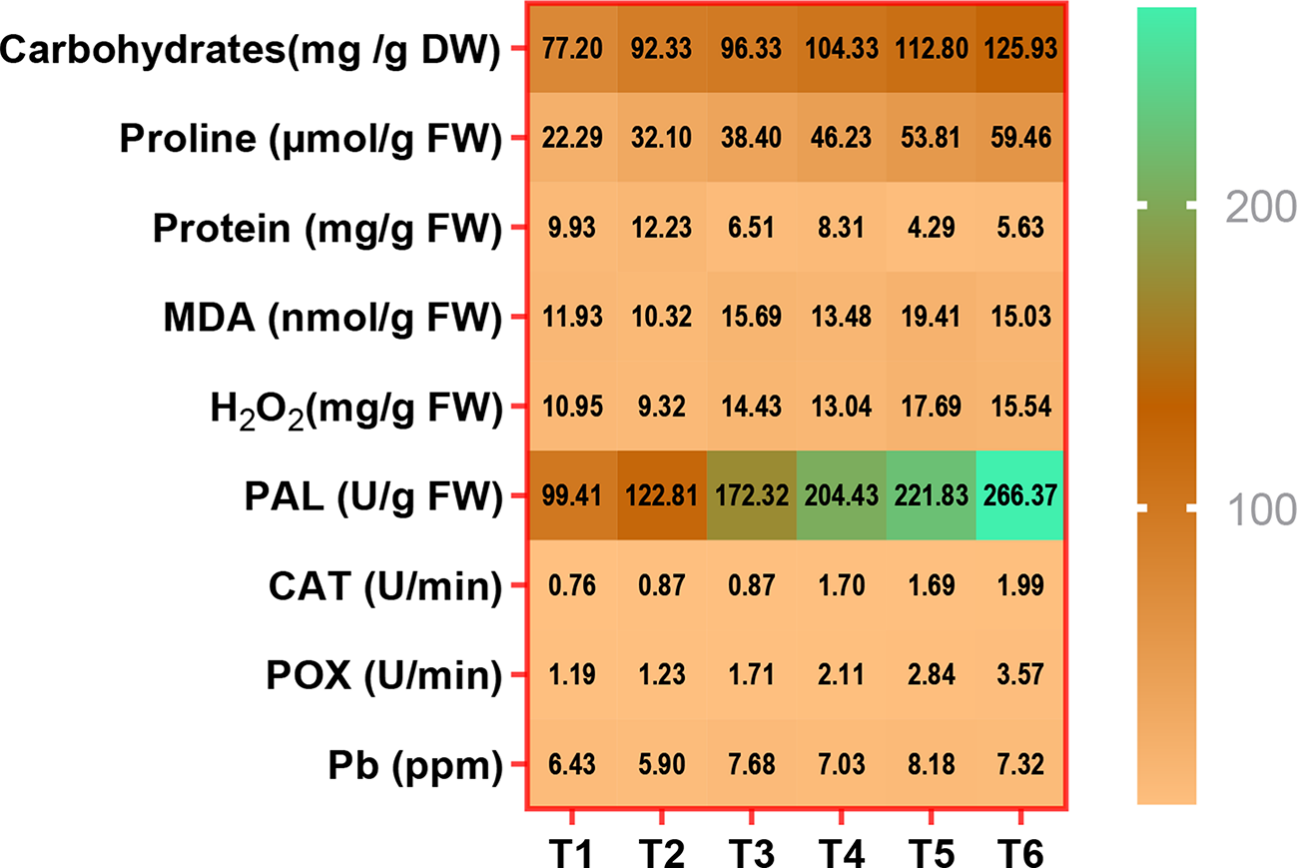
Heatmap constructed between different measured parameters. T1, T3 and T5 represent *V. faba* plants grown under 0, 50 and 100 ppm Pb concentrations, while T2, T4 and T6 represent *V. faba* plants foliar sprayed by Chs under 0, 50 and 100 ppm Pb concentrations

When faba bean plants were exposed to 50 and 100 ppm of Pb, their Pb concentration increased dramatically. Data graphed in Fig. 5 revealed an increase of 19.45 and 27.23 of Pb in faba bean shoot at 50 and 100 ppm compared to control. Although Pb is non-essential, plants absorb it easily leading to an increase in its concentration. The plants take up the Pb from the atmospheric air through cellular respiration, where the large surface area of plant leaves permits the absorption of Pb ions from polluted air via cuticle and stomata causing chlorosis in leaves [100]. In line with our results, Ansari et al. [2] reported that Pb concentration of shoot and root of *Lallemantia iberica* increased with increasing Pb levels. Nevertheless, interesting results were obtained with Chs treated plants where the foliar application of Chs decreased the Pb concentration in plants as shown in Figs. 5 and 6. Under Pb stress conditions, Chs application was successful (*p* ≤ 0.05) and led to about 8.46% (at 50 ppm Pb) and 10.43% (at 100 ppm Pb) decreases in comparison to those of Pb treated plants only (Fig. 5). Zong et al. [14] reported that foliar application of different molecular weight Chs alleviate toxic effects of Cd in a hydroponically grown edible rape (*Brassica rapa* L.). Furthermore, consistent with this study’s findings, Chs treatment decreased the accumulation of Cd in radish [101] and Ni in soybean [69] and this may be due to that Chs can form complexes with a series of heavy metals because of the presence of amino and hydroxyl groups [102] so, it reduces the absorption and transfer of these metals to plants.

#### Genomic DNA profiling by ISSR in faba bean shoots as affected by Chs application under Pb stress

**Fig. 7:**
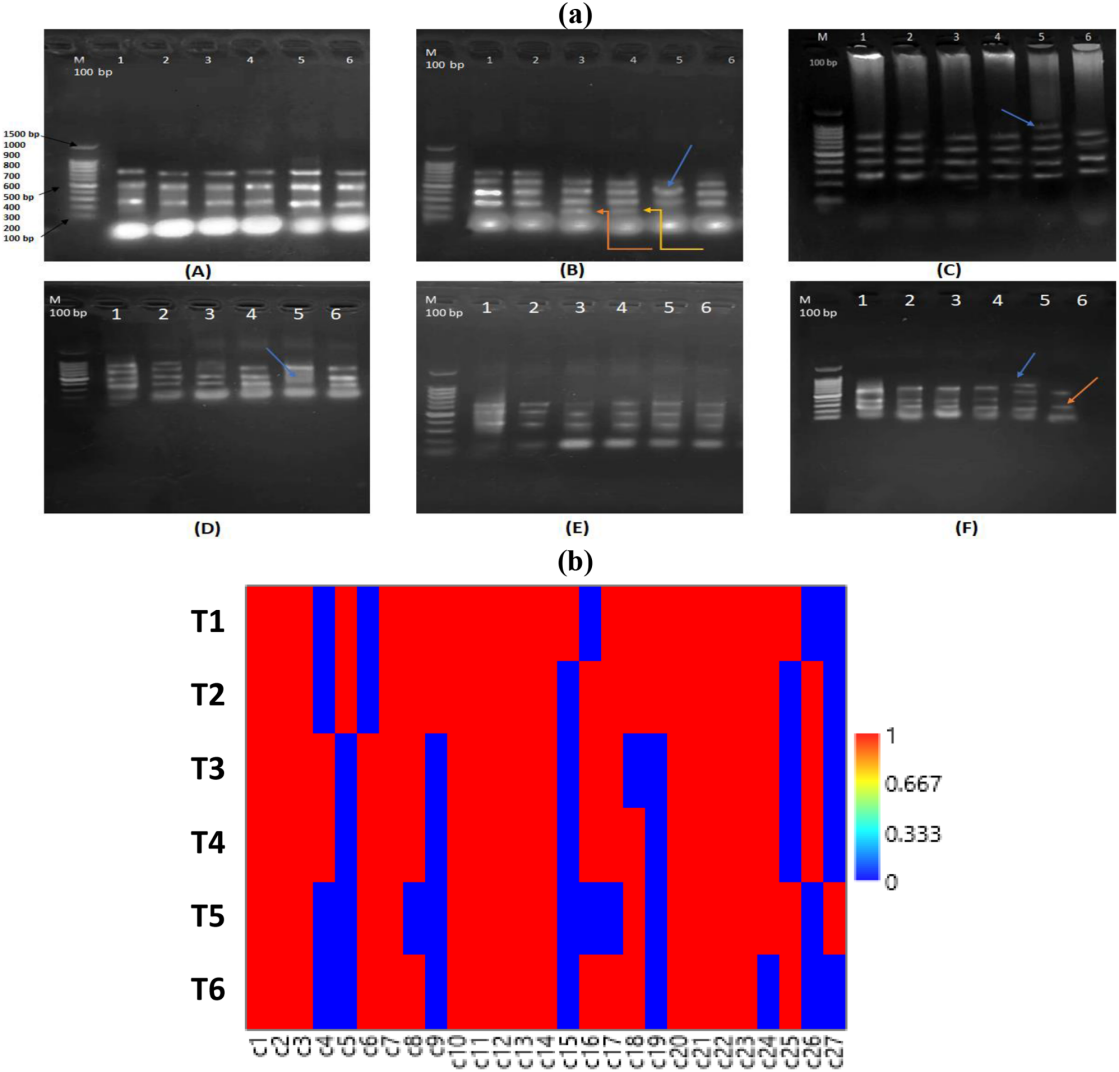
(**a**) The Inter Simple Sequence Repeats (ISSR) products of genomic DNA extracted from leaves of *V. faba* plants using six primers, where arrow indicating appearance or disappearance of bands (**b**) Matrix plot constructed using the Past-pc showing band number of amplified DNA markers produced by ISSR marker, C1-C3 for (AC)8GG, C4-C9 for (CTC)6, C10 to C14 for (TG)8AA, C15-C18 for (AG)8T, C19-C21 for (TG)8AA and C22-C27 for (CA)8T. T1, T3 and T5 represent *V. faba* plants grown under 0, 50 and 100 ppm Pb concentrations, while T2, T4 and T6 represent *V. faba* plants foliar sprayed by Chs under 0, 50 and 100 ppm Pb concentration

Inter simple sequence DNA (ISSR) has shown effective in verifying genetic homogeneity in plants exposed to various kinds of heavy metals and abiotic stressors [103–106]. This technique was performed to evaluate the effect of different concentration of Pb and Chs on the genetic material of faba bean cultivated plants in comparison to control. About ten primers were used for evaluating the genetic polymorphism in faba bean fresh leaves as shown in Table (2), only six primer give reproducible bands appeared in (Fig. 7- a and b). The code and sequences of the ten ISSR primers were listed in Table (2).

In total, one hundred and twenty-two (122) amplified DNA bands were scored after using the six primers. These six primers generated a total number of 64 mono-and polymorphic DNA bands with a high polymorphism value of 95.31%. The highest polymorphism values of 100% were scored at primer- M2NL-4, M2NL-5, and M2NL-6. In contrast, the lowest polymorphism value of 66.60% was scored at primer-ISSR-5 and ISSR-6. These polymorphisms are based on specific random sequences, the sort of DNA bands (unique, non-unique, and monomorphic), their intensity, and their length, ranging between 0.2 and 0.9 Kbp. ISSR-5amplified the most bands (14), whereas ISSR-2 amplified the fewest bands (7) and this agreed with Labra et al. [107] and Al-Qurainy [108] where they found that ISSR analysis indicated that heavy metals showed genotoxicity at high concentrations induced DNA changes in different target sequences.

**Fig. 8:**
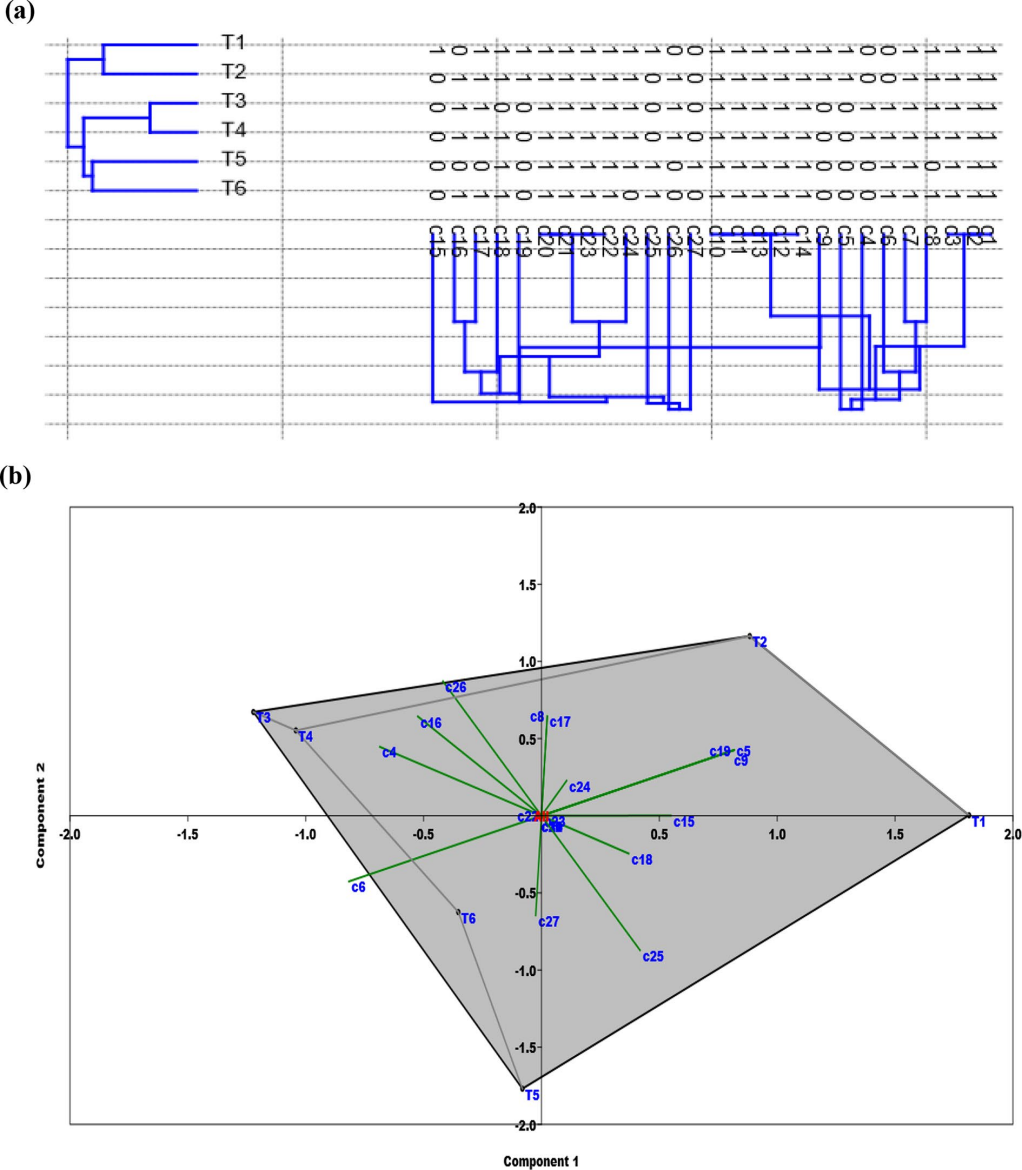
(**a**) UPGAMA Distance tree based on Euclidean, constructed using the Past-pc, showing the genetic distance between different treatments. (**b**) PCA constructed using the Past-pc showing band number of amplified DNA markers produced by ISSR marker. C1-C3 for (AC)8GG, C4-C9 for (CTC)6, C10 to C14 for (TG)8AA, C15-C18 for (AG)8T, C19-C21 for (TG)8AA and C22-C27 for (CA)8T. T1, T3 and T5 represent *V. faba* plants grown under 0, 50 and 100 ppm Pb concentrations, while T2, T4 and T6 represent *V. faba* plants foliar sprayed by Chs under 0, 50 and 100 ppm Pb concentrations

The PAST software-based clustering analysis utilizing the Euclidean equation generated two significant database-based clusters, I and II (Fig. 8a**).** The first (I) cluster was split into two subclusters, T1 and T2, the second cluster (II) comprised four treatments, T3 and T4 in same group and T5 and T6 in another group. PCA scatter plot constructed using the PAST-pc 4.2 software showing the relationships among the examined treatments based on ISSR fingerprinting polymorphism (Fig. 8b). The examined treatments were differentiated into three subgroups by the PCA scatter plot, identical to their separation in the clustering analysis.

Genetic similarities were computed using the Kulczyuski-similarity index value. The maximum genetic similarity between both 50 mM pb and 0.1% Chs + 50 mM Pb was 0.97, while the minimum genetic similarity between 0.1% Chs and 100 mM Pb was 0.75 (Table 5). The results showed that new DNA bands are emerging in the ISSR profile, and that the absence of typical bands can be interpreted as a mutation. This is most likely the result of genetic variation produced by rearrangements or DNA damage [106, 109]. ISSR primers led to differences in the amount of variation observed among genotypes, and as a result of genetic drift caused by Pb toxicity, it is anticipated that genetic diversity will decline over time [110].

**Table 5:** Similarity index calculated by Kulczyuski index for ISSR primer code in the studied samples, values represented as similarity index %

Samples	Control	0.1% Chs	50 ppm Pb	0.1% Chs + 50 ppm Pb	100 ppm Pb	0.1% Chs + 100 ppm Pb
**Control**	1					
**0.1% Chs**	0.90909091	1				
**50 ppm Pb**	0.76363636	0.85909091	1			
**0.1% Chs + 50 ppm Pb**	0.79112554	0.88419913	0.97619048	1		
**100 ppm Pb**	0.80808081	0.75757576	0.79166667	0.82539683	1	
**0.1% Chs + 100 ppm Pb**	0.83373206	0.83373206	0.87236842	0.90225564	0.86549708	1

## Conclusion

The study concludes that excess Pb severely affects plants’ morphology and physio-biochemistry, leading to the deficiency of food security and economic losses. The results demonstrated that with increasing Pb concentrations, there was a reduction in growth, pigments and proteins contents. In the same time, a significant increase in the stress markers, both MDA and H_2_O_2_, was observed under 50 and 100 ppm Pb. The self-defense system of the plant is not adequate to reduce the negative effects of Pb. The foliar application of Chs improves the tolerance of faba bean plants to Pb stress by significantly improving the faba bean growth, pigment fractions, protein, carbohydrates, reducing MDA and H_2_O_2_ contents and decreasing Pb concentrations. Pb mitigation effects by Chs are probably related with the activity of antioxidant enzymes, PAL and proline. Also, ISSR analysis outlines distinct genetic clusters and subgroups corresponding to different treatments and the genetic similarity reflecting the variability in genetic response among treatments. Based on the obtained results, we recommended spraying faba bean plants with Chs under Pb stress to enhance plant physiological, biochemical and molecular parameters and offset this stress. In the future, we looking forward to investigate the specific genetic mechanisms underlying heavy metal tolerance in faba bean plants, including identifying candidate genes and pathways involved in stress response and also explore the potential of heavy metal-tolerant varieties for phytoremediation purposes, including their ability to accumulate and detoxify heavy metals from contaminated soils.

### Electronic supplementary material

Below is the link to the electronic supplementary material.

Supplementary Material 1

## Data Availability

The relevant datasets supporting the results of this article are included within the article. There is no sequencing data generated in the current study.

## Abbreviations

CAT: Catalase
Chl: Chlorophyll
Chs: Chitosan
DW: Dry weight
FW: Fresh weight
ISSR: Inter simple sequence repeat
MDA: Malondialdehyde
PAL: phenylalanine ammonia lyase
POX: Peroxidase
ROS: Reactive oxygen species
SE: Standard error
