# Supplementary figures and images for: Offsetting pb induced oxidative stress in Vicia faba plants by foliar spray of chitosan through adjustment of morpho-biochemical and molecular indices
Source: BMC Plant Biol. 2024 Jun 14;24:557. doi: 10.1186/s12870-024-05227-w (PMC11177494; doi:10.1186/s12870-024-05227-w)

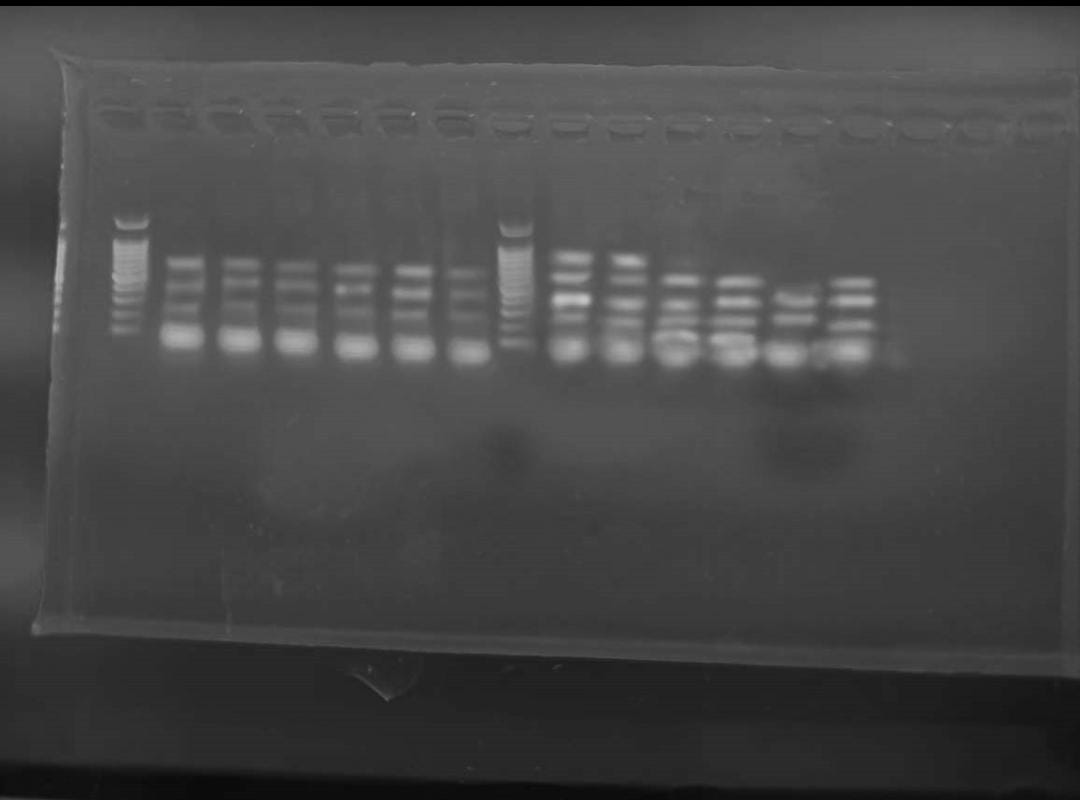


**Supp. 1- Fig 7A (a,b)**


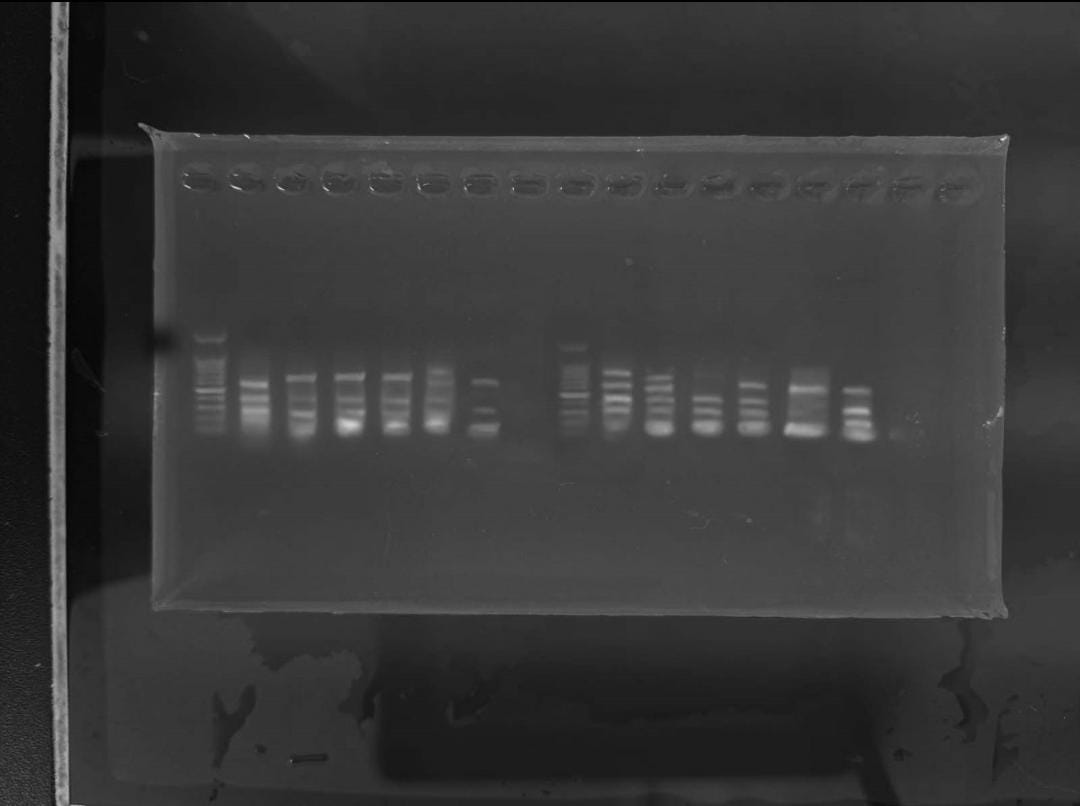


**Supp. 2 –Fig 7A (d,f)**


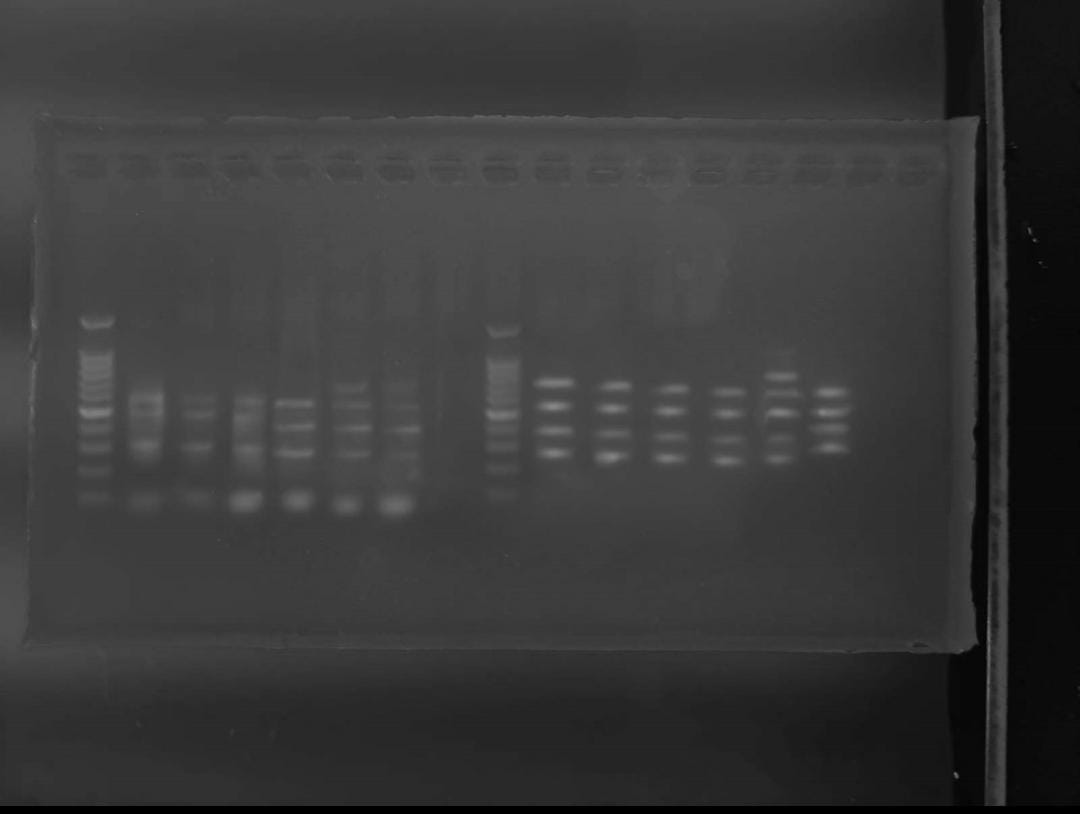


**Supp. 3 -Fig 7A (E,C)**

Supplement: Supplementary file 1 — Supplementary Material 1 [file 12870_2024_5227_MOESM1_ESM.docx]
